# Characterization of Neonatal Seizures in a Large Well-defined Multicenter Cohort of a Tertiary Neonatology Center in Germany

**DOI:** 10.1055/a-2710-4474

**Published:** 2025-10-16

**Authors:** Verena Kraus, Ulrich Schatz, Marcus Krüger, Franziska Krampe-Heni

**Affiliations:** 1 Department of Pediatrics, German Center for Child and Adolescent Health (DZKJ), TUM School of Medicine and Health, Technical University of Munich, Munich, Germany; 2 Faculty of Medicine, Chair of Social Pediatrics, Technical University of Munich, Munich, Germany; 3 Institute for Human Genetics, TUM School of Medicine and Health, Munich, Germany; 4 Department of Neonatology, Children's Hospital Munich Schwabing and Harlaching, Munich, Germany

**Keywords:** neonatal seizures, seizures, perinatal care

## Abstract

**Introduction:**

Prevalence of seizures is 1 to 5/1,000 neonates. The most common causes of neonatal seizures are hypoxic–ischemic encephalopathy (HIE), vascular events (hemorrhages, stroke), and infections. We assessed prevalence and etiology of seizures defined according to the recent Brighton and International League of Epilepsy (ILAE) criteria in a neonatology monocenter cohort.

**Methods:**

In a retrospective cross-sectional cohort study of all 12,154 neonates born in our three maternities from January 1, 2022 to December 31, 2023 seizures were categorized by frequency, etiology, risk profile, semiology, and EEG. A total of 19 neonates (male: *n* = 11 [57.9%]; full-term: *n* = 11 [57.9%]; preterm very low birth weight [VLBW]: *n* = 6 [31.6%]; preterm >1,500 g birth weight: *n* = 2 [10.5%]) were identified.

**Results:**

In 19/12,154 neonates, seizures were confirmed by application of the ILAE criteria. Preterm VLBW was found in 174 neonates with birth weight <1,500 g. Seizure incidence was 1.6/1,000 in all neonates and 3.4% in VLBW infants. HIE was the most frequent etiology in term infants (30.8%), followed by vascular events in preterm >1,500 g and term infants (30.8%). Vascular events were the most common cause in preterm VLBW infants (83.3%). Whole exome sequencing (WES) was performed in four cases (21.1% of neonates with seizures).

**Discussion:**

Incidence of neonatal seizures in our center is in the lower range and leading seizure etiologies are comparable to the literature. Early recognition of neonatal seizures including the detection of electrographic-only seizures and early WES to identify rare genetic defects possibly offering tailored treatment options have the potential to further raise the standard of neonatal care and improve neurodevelopmental outcome.

## Introduction

Neonatal seizures have an estimated frequency of 15/1,000 live births (LB). 1 They are defined in terms of timing of occurrence: in term newborns (1.10–3.5 cases/1,000 LB), in the first 4 weeks postpartum, and in premature babies (10–14.28 cases/1,000 LB), in the first 44 weeks of gestational age. 2 3 4 5 6 Large unselected cohorts show that the frequency of neonatal seizures in term babies is 0.5% and in preterm babies 22.2%, and correlate inversely with the level of prematurity. 7 The International League Against Epilepsy (ILAE) has developed a generally accepted terminology, classification, and diagnostic and therapeutic recommendations for neonatal seizures. 8 9 Neonatal seizures are usually provoked. The most common causes of neonatal seizures overall are hyp-oxic–ischemic encephalopathy (HIE), vascular events (hemorrhages, stroke), and infections. 1 Other causes include acute metabolic disorders such as electrolyte imbalances and hypoglycemia or inborn errors of metabolism, neonatal epilepsy syndromes, and malformations. 10 In term neonates, the most common cause is HIE; other causes include neonatal stroke or hemorrhage, cortical malformations, metabolic disorders, and infections. 9 11 In premature babies, the most common cause is intraventricular hemorrhage, followed by infection. 12 13 According to the classification of the ILAE Neonatal Seizures Task Force and a retrospective analysis of neonatal seizures in 40 neonates, all regarding the semiology of the seizures and the most likely etiology, neonatal seizures with a genetic cause often present as sequential semiology clonic, myoclonic, or tonic, irrespective of the nature. 8 14 A clear correlation of clinical features with a specific genetic cause is currently only possible in a few cases. 15 16 17 In recent years the incidence of neonatal seizures was constantly rising because of the better detection of electrographic seizures only, especially in developed countries. The incidence was decreasing in poor countries because of a higher level of care. 18 In parallel the early diagnosis of developmental epileptic encephalopathy (DEE) is made by broader accessibility to whole exome sequencing (WES). 1 19 20 21 22 23 24 Early seizure therapy and prevention of status epilepticus lower the risk of neuropsychological sequelae. 6 25 The early differentiation between acute symptomatic and DEE is crucial for prognosis. 26 A study conducted in 2017 found 15% of seizures in neonates were attributed to neonatal epilepsy syndromes with or without brain malformations. 27 Electrographic-only seizures are the most prominent seizure type in preterms making aEEG monitoring inevitable. However, aEEG is also limited in sensitivity for short seizures. This makes sufficient monitoring a challenge for most neonatal intensive care units (NICUs). 26 In addition, children with epilepsy after acute apparently structural symptomatic neonatal seizures show a higher prevalence of genetic variants than patients without subsequent epilepsy. 28

The aim of the study was to assess the incidence and characterize the levels and seizure types in neonates according to the ILAE criteria at our center during the years 2022 and 2023.

## Methods

### Study Design

The present study is a retrospective cross-sectional cohort study. Our neonatal center is one of the largest tertiary perinatal centers in the region comprising three maternities. Preterm very low birth weight (VLBW) infants are defined as <1,500 g birth weight. Total number of neonates born from January 1, 2022 to December 31, 2023 were analyzed. Newborns are defined as infants within 44 weeks of gestation. Only neonates who presented with a seizure during the neonatal period and were diagnosed and treated at our neonatal center were included. The diagnosis of a neonatal seizure was made according to the ILAE criteria and classified by the Brighton criteria (levels 1–5) into the different levels of certainty. 8 Suspected seizures in the clinical database were defined by the ICD10 code P90 for neonatal convulsions.

### Recruitment Measures

We searched applying the ICD10 code P90 “Convulsions in newborns” in our standard patient data management system. The inclusion criteria were then checked based on the children's discharge report. If the criteria were met, they were included in the study. A total of 31 patients with the above-mentioned ICD code were identified, 3 children were listed twice in the database because they had been transferred between NICU wards and were therefore registered in the database in multiple locations. Electrophysiological, imaging, and clinical data were evaluated for final seizure classification. EEG was evaluated by a neuropediatrician with additional qualifications in the evaluation of EEG. In emergency situations EEG could not be performed because of the necessity of acute lifesaving interventions in *n* = 2 neonates. All babies underwent ultrasound scan of the brain, and 12 babies also received an MRI of the brain; in one baby a CT scan of the brain was performed due to an emergency situation. Out of the 28 babies 9 had to be excluded because the event was finally not classified as a seizure (Brighton criteria level 5): 7 babies were finally diagnosed with benign neonatal (sleep) myoclonus or pulmonary aspiration events. One child had irritability due to drug withdrawal of maternal polymedication with antidepressants and opioids. In one child there was a miscoding with the mother herself presenting with postnatal convulsions. An epileptologist made the final seizure classification. Finally, a total of 19 children were included in the data analysis with a diagnosis of neonatal convulsions. The criteria were met by 174 neonates with preterm VLBW of <1,500 g birth weight and/or <32 weeks of gestation.

### Statistics

The evaluation was performed by means of descriptive statistics (absolute and relative frequencies) in SPSS version 28.01.1.

## Results

### Demographic Data

**Fig. 1:**
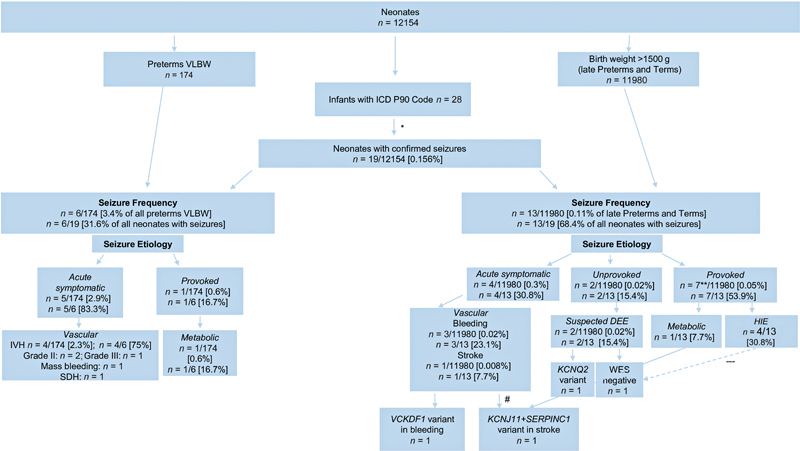
*: 9 neonates with other diagnoses (level 5 of Brighton criteria): *n* = 1 neonatal withdrawal syndrome, *n* = 1 maternal seizure, *n* = 4 benign neonatal myoclonus/sleep myoclonus, *n* = 1 incorrect controlling code, aspiration after feeding, *n* = 1 prophylactic diagnostics because of suspected seizure in twin sister. One patient was reclassified after the negative whole exome sequencing (WES) as supposedly hypoxic–ischemic encephalopathy (HIE), not included in the total number of HIE. **: One patient was not a term neonate but a twin with fetofetal transfusion syndrome born at 32 + 4 weeks of gestation and 1,740 g in weight, who formally fulfilled the HIE criteria defined for term neonates, but was excluded from the formal HIE cohort as discussed in the text. #: Two etiologies in the same patient: first seizure due to hypoglycemia and the second one due to stroke. DEE, developmental epileptic encephalopathy; IVH, intraventricular hemorrhage; SDH, subdural hemorrhage; VLBW, very low birth weight (<1,500 g birth weight and/or <32 weeks of gestational age).

We analyzed all 12,154 neonates born at our center during the years 2022 and 2023. For demographic data, see Table 1 . About 28% of all newborns in the city and nationwide 0.8% of all newborns are born in these three maternities. *N* = 174 were preterm VLBW infants. 28 of those with the diagnosis of neonatal seizures were identified in the database. Because of other diagnoses than seizures 9 had to be excluded (see Fig. 1 for details). A total of 19 neonates with seizures were further analyzed according to maturity and etiology of seizures. Seizures occurred in 6 preterm VLBW infants and 11 term infants and 2 preterm infants with >1,500 g birthweight (see Fig. 1 and Table 1 for details). Of these 19 neonates the overall sex distribution was 57.9% male with a female preponderance in preterms and a male preponderance in term newborns (see Table 1 ). The median gestational age was 34.99 ± 6.60 weeks (range: 24 + 4–42 + 1 weeks). The median birth weight was 2,368.95 ± 1,229.68 g (range: 550–3,850 g); the APGAR score was lower in the preterm cohort on average. All of these neonates were inborn in one of our three maternities.

**Table 1:** Description of the sample

Parameter	Preterms	Terms	Total
Classification	*N* = 8 [42.8%]	*N* = 11 [57.9%]	*N* = 19 [100%]
Sex	Male: *N* = 3 [37.5%]; female: *N* = 5 [62.5%]	Male: *N* = 8 [72.7%]; female: *N* = 3 [27.3%]	Male: *N* = 11 [57.9%]; female: *N* = 8 [42.8%]
Gestational age	<28 weeks: *N* = 4 28–32 weeks: *N* = 2 32–37 weeks: *N* = 2	>37 weeks: *N* = 11	m = 34,99 ± 6,60 weeks (range: 24 + 4–42 + 1 weeks)
Birth weight	<1,000 g (ELBW): *N* = 5 1,000–1,499 g (VLBW): *N* = 1 1,500–2,500 g (LBW): *N* = 2	2,500–3,000 g: *N* = 2 3,000–4,000 g: *N* = 9 >4,000 g: *N* = 0	M = 2,368.95 ± 1,229.68 g (range: 550–3,850 g)
Umbilical cord pH	<7.0: *N* = 2 7.0–7.09: *N* = 0 7.1–7.19: *N* = 1 >7.2: *N* = 4 not known: *N* = 1	<7.0: *N* = 4 7.0–7.09: *N* = 7.1–7.19: *N* = 1 >7.2: *N* = 3 not known: *N* = 3	m = 7.13 ± 0.25 (range: 6.70–7.43) [ *n* = 14]
APGAR score at 5 min	0–3: *N* = 0 4–7: *N* = 4 8–10: *N* = 3 not known: *N* = 1	0–3: *N* = 2 4–7: *N* = 4 8–10: *N* = 5	M = 7.0 ± 2.33 (range: 3–10) [ *n* = 18]
Death before discharge	*N* = 2	*N* = 0	*N* = 2

Note: m = mean +/− STD.

### EEG Recordings

Conventional EEG was performed on *n* = 15 neonates; *n* = 2 neonates were too instable to perform EEG in the acute situation and died due to acute complications. One neonate had a seizure in the context of hypocalcemia and was subsequently always neurologically unremarkable and therefore did not receive a conventional EEG in the course of the disease. aEEG was performed in *n* = 16 neonates, *n* = 5 of which were preterm and *n* = 11 term born.

### Incidence of Seizures

The incidence of neonatal seizures was 0.16% (1.6 in 1,000 live births). Of *n* = 174 preterms in the high-risk category (<1,500 g birth weight and/or <32 weeks of gestation) *n* = 6 were diagnosed with seizures.

### Etiology of Seizures in the Preterm VLBW Cohort

In most cases the etiology in the preterm VLBW cohort was attributed to intraventricular hemorrhage (IVH): Four babies of the preterm VLBW cohort had IVH, and one baby had a subdural hemorrhage. According to data from the German Neonatal Network, our perinatal center does not have a statistically higher incidence of cerebral hemorrhages in neonates compared with other centers ( https://perinatalzentren.org ). About 50% of these IVHs were grade 2. All premature neonates who had a seizure due to a cerebral hemorrhage were preterm VLBW infants ( *n* = 4). Provoked seizures with 16.7% were the second most frequent cause of seizure in preterm VLBW infants.

### Etiology of Seizures in the Cohort of Neonates with >1,500 g

In term infants and preterms with birth weight >1,500 g provoked seizures at 53.9% were the most frequent seizure etiology. Five term neonates were diagnosed with HIE according to standard diagnostic criteria. One neonate was a twin with fetofetal transfusion syndrome born at 32 + 4 weeks of gestation and 1,740 g weight and fulfilled the HIE criteria defined for term neonates. No other provoking factor for seizures was identified. (The four term neonates received hypothermia; the premature infant did not receive hypothermia because of immaturity.) Acute symptomatic seizures occurred about twice as often as unprovoked seizures accounting for 30.8 and 15.4%, respectively.

### WES in the Context of Seizures

In total WES was performed in four patients. Genetic analysis by WES because of suspected neonatal epilepsy syndromes or DEE was performed in 15.4% of the cases. In provoked and acute symptomatic etiologies WES was also performed in the same number of cases. A genetic diagnosis could be established in three patients (75% of the cases). No signs of neonatal infection or malformations as a cause of seizures were found. Seizures in these term born neonates were all present during the first 72 hours after birth, which is a criterion for acute symptomatic or provoked seizures. However, only in suspected DEEs, seizures were sequential. One patient showed two acute events. The first seizure was provoked by a metabolic change; the second seizure was acute symptomatic because of a vascular event. Interestingly this patient showed two pathogenic genetic variants despite sequential seizures. Every variant led to an acute event at different times after births and so was the reason for the seizure. The *KCNJ11* variant led to hyperinsulinism and hypoglycemia and provoked the first seizure within the first 72 hours after birth. The second pathogenic genetic variant was *SERPINC1* , which leads to thrombophilia. The pathogenic variant was the reason for a deep venous thrombosis in the mother during the time of immobilization after birth and for a stroke in the child, as the mode of inheritance is autosomal dominant. The stroke led to the acute symptomatic seizure at a time point, where blood sugar levels were regularly monitored and within the normal range. So, in our cohort in two cases rapid WES was performed not for suspected DEE, but because of a severe vascular event. In the first case, stroke of the middle cerebral artery and thrombosis in the mother with immobilization after birth led to the suspicion of a coagulation disorder. In the second case, a mass bleeding and dysmorphic stigmata led to genetic reanalysis. The clinical exome in this term born infant with the mass bleeding was already negative, but the new combination of the symptoms (suspected bleeding disorder and malformations with midface hypoplasia and micromelia) led to the detection of a pathogenic *VCKDF1* variant. Two children presented with therapy refractory seizures beyond the time frame for acute symptomatic seizures and exhibited sequential seizures. So, they were supposed to have DEE. In these two cases in one DEE a *KCNQ2* variant was diagnosed; in the other patient seizures were first therapy refractory, but then stopped and all AEDs could be withdrawn without any recurrence of seizures or signs of DEE. Seizures were reclassified most likely as acute symptomatic due to HIE.

### Etiology of Symptomatic Seizures

Overall, in all neonates with acute symptomatic seizures vascular events occurring in 9/19 were the most common etiology followed by HIE. HIE at 30.8% was the most common cause of neonatal seizures in term neonates (see Fig. 1 ). Four preterm infants with vascular events had hemorrhage; one had chronic subdural effusion diagnosed after the acute phase. Three term neonates had hemorrhage; one had a stroke. Metabolic reason was the second most common cause in preterm VLBW infants accounting for 0.6%. DEE was the least common cause at approximately 15.4%. None had infection as cause of seizure. One child with infection had a provoked seizure due to hyponatremia. Epilepsy was more frequent in term infants during the study interval. Late-onset epilepsy deriving from an acute symptomatic course later in life was not assessed. One justifying etiology was found in 18 of 19 patients; two justifying etiologies for two different seizure episodes (hypoglycemia and stroke) in 1 of 19 neonates.

### Diagnostic Certainty of Seizures

We then analyzed the level of diagnostic certainty in our cohort. Six (31.5%) seizures could be classified as level 1, seven (36.8%) as level 2a or 2b, four (21.1%) as level 3, and two (10.5%) as level 4. Nine babies were classified as level 5; so no seizure but an alternative diagnosis (4/9 presenting with benign sleep myoclonus). The most frequent seizure type was clonic with *n* = 12 (63.2%), followed by autonomic and myoclonic. The least frequent seizure type was tonic. *N* = 0 seizures were electrographic only. Sequential seizures were only observed in diagnosed DEE and suspected DEE with negative WES. One seizure was not specified in semiology (see Table 2 for details).

**Table 2:** Seizure classification, semiology, and etiology according to ILAE criteria, multiple mentions with seizure types are possible

Level	Semiology	Etiology	Gestational age
1	Clonic and autonomic	Provoked (HIE) *n* = 1	40 + 3 weeks
	Myoclonic	Provoked (HIE) *n* = 0	
	Myoclonic–autonomic	Unprovoked (epileptic encephalopathy without genetic diagnosis, reclassified provoked likely HIE) *n* = 1 Provoked with bleeding *n* = 1	40 + 1 weeks
			32 + 5 weeks
	Clonic	Unprovoked ( *KCNJ11* + *SERPINC1* ) with stroke ( *n* = 1) and *KCNQ2* ( *n* = 1); acute symptomatic (bleeding), *n* = 1	38 + 6 weeks
			38 + 6 weeks
			41 + 2 weeks
2a	Semiology not specified	Provoked ( *VCKDF1* ) with bleeding *n* = 1	39 + 0 weeks
	Clonic	Provoked (HIE) *n* = 4; provoked with bleeding *n* = 1	32 + 4 weeks; 39 + 5 weeks; 39 + 6 weeks; 41 + 5 weeks
			27 + 2 weeks
2b	Clonic	Provoked (metabolic) *n* = 1; acute symptomatic (bleeding) *n* = 1	41 + 3 weeks
			24 + 6 weeks
3	Clonic	Acute symptomatic (bleeding) *n* = 1	25 + 3 weeks
	Autonomic	Acute symptomatic (bleeding) *n* = 3; provoked *n* = 1 (metabolic)	42 + 1 weeks; 28 + 6 weeks; 24 + 4 weeks
			28 + 1 weeks
4	Tonic	Provoked (HIE) *n* = 0	

Abbreviations: HIE, hypoxic–ischemic encephalopathy; ILAE, International League of Epilepsy.

Electroclinical seizures were found in nine neonates ( *n* = 3 preterm and *n* = 6 term, of which *n* = 4 had an HIE). Electrographic seizures were common in HIE (66%); clonic seizures were found in vascular etiologies, especially in stroke, where focal clonic seizures of the contralateral side are provoked.

## Discussion

We present a systematic retrospective analysis and comprehensive overview regarding the incidence, etiology, seizure semiology, and electrophysiology of neonatal seizures in a perinatal center with highest level of care in Germany—representative of around 0.8% newborns nationwide. The seizures in neonates during the years 2022 and 2023 were classified using the Brighton criteria. 26

### Preterm Infants

The frequency of 1.6/1,000 is in the lower range compared with the literature and corresponds to the incidence found in other studies of 1.2/1,000 live births. 29 We have at our center the highest standard of care, minimizing additional risk factors for seizures especially in preterm VLBW infants. This is reflected in the absence of infection and only two patients with bleedings grade >2 in the preterm VLBW cohort. The highest population-based incidence was reported to be 5/1,000 live births. 1 The incidences in the literature vary because of two reasons: First, in countries with lower medical standards more neonates present with seizures due to infections, metabolic reasons, and HIE. 30 Second, in epilepsy centers more electrographic-only seizures with higher level of certainty according to the Brighton criteria are detected especially in preterm neonate with risk factors. 26 Infants with risk factors beyond HIE are monitored with continuous EEG, which increases the detection of electrographic-only seizures. 25 In line with this preterm VLBW infants have in recent studies have reported seizures in 8.8% of cases, aggravating with risk factors mainly due to prematurity. 31 IVH and asphyxia represent the major etiologies for seizures in preterm VLBW and term infants respectively in our cohort. 3 31 32 Recently another center of highest neonatal care in the south of Germany published a study with a cohort of 34 preterm infants with IVH grades 2 to 4 that presented with seizures in 52.9% of cases, 13 which is similar to our findings. These complication rates are within the lower range in comparison to all neonatal centers in Germany. However, bleeding as a seizure cause might be overrepresented in our study. A bleeding detected during routine ultrasound monitoring on days 1, 3, and 7 in preterm infants is twice performed during the critical period for acute provoked seizures. A detected bleeding might raise the suspicion for clinical seizures, which are then confirmed by EEG. 33 Further etiologies in preterm infants are also dependent on vulnerability according to gestational age. 11 A Korean population based study showed that in the entire group of preterm infants congenital anomaly, delivery room resuscitation, maternal histologic chorioamnionitis, surfactant use, hypotension within 1 week of life, sepsis within 14 days after birth, and meningitis and NEC of at least stage 2 increased the seizure risk. 31 In preterm VLBW infants monitoring detected seizures in 20% of infants. 31 Continuous seizure monitoring as introduced by the 2023 guidelines had not been implemented yet for at-risk neonates in our center in the two analyzed years, but is now set up. As 14% of seizures in preterm and 24% of seizures in term neonates are electrographic only, introduction of continuous seizure monitoring independent of ultrasound findings, but adapted to other risk cohorts especially infection and level of immaturity, will presumably rise the incidence of neonatal seizures and lower the proportion of IVH. 3 9 28 34 Especially in preterm infants with risk factors the early detection of seizures is important, because they show an impaired neurodevelopmental outcome. 13 32 Term infants benefit in the developed countries from the increased standard of neonatal care including hypothermia and close monitoring for metabolic imbalances and infection as well as aEEG monitoring. 35

### Term Infants and HIE

In our cohort pure electrographic seizures are detected in term newborns with HIE, because routine aEEG monitoring is already implemented according to the best standard practice protocols. However, all neonates with aEEG monitoring also showed clinical seizures in our study. It is possible that the electrographic monitoring lowers the threshold of clinical examination for seizure signs in the staff and so subtle clinical seizures are also detected more often. It is known that strict hypothermia protocol lowers the incidence of seizures due to HIE by 50%. 36 In line with this explanation one late preterm infant fulfilling the criteria of HIE for term neonates and where hypothermia was not applicable because of immaturity, developed seizures. Although HIE is still the most common cause in term neonates in our study, as in the literature genetic etiologies are also increasingly detected. 24

### Genetic Evaluation

The early detection of a genetic cause has the potential to reduce invasive and costly procedures especially in instable patients in tertiary neonatal and intensive care units 37 and offer tailored therapy early in the disease course. The current recommendation is to carry out a genetic diagnosis in the absence of a structural lesion (such as bleeding, stroke, or infection) or in the presence of sequential seizures, epileptic spasms, or tonic seizures. 14 38 In 21.05% of cases with neonatal seizures at our center WES was performed. In 30.8% of term or preterm >1,500 g birth weight neonates with seizures WES enabled an etiological classification in 75% of cases. Diagnoses include channelopathy affecting epilepsy and metabolism, syndrome with malformations, exclusion of developmental epileptic encephalopathy, and thrombophilia. The frequencies correspond to other studies but were overall rare. This could be, on the one hand, due to the fact that we analyzed only 2 years of our inpatient births. On the other hand, we are not a specialized epilepsy center with a selection bias for therapy refractory cases referred from another center. 20 27 In fact some neonatal seizures look like a secondary genesis, but are genetic. 38 We show this in patients with vascular causes; in the literature this is demonstrated for patients with HIE. 24 39 Overall, these indications are still rare but will likely be increasingly diagnosed with the broader availability of WES, especially in electroclinical sequential seizures detected by continuous seizure monitoring and therapy refractory seizures with the need for early tailored causal therapy or palliative care in the future. 6

The limitations of our study include the presentation of term or late preterm neonates within the first 4 weeks of life after discharge to another neonatology unit and so the number of seizures in beginning neonatal epilepsies, especially DEE, can be underestimated. Also discharge directly after birth could have underestimated additional provoked seizures within the first 72 hours of life. EEG monitoring was not performed uniformly. aEEG was considered in cases of clinical seizures and was done in HIE according to standard protocols. Electrographic seizures, especially in preterm infants, are therefore underestimated in our cohort, as clinical signs or HIE led to aEEG/cEEG confirmation or exclusion of clinical suspicion. With the introduction of routine EEG monitoring in tertiary neonatal centers according to the neonatal guidelines for neonates at risk, which are based on the international consensus paper, we will close this gap in the future. 9 The first year of life shows the highest incidence of epilepsy following acute neonatal seizures and decreases gradually within the first 20 years of life. 40 Indications for WES beyond the suspicion of DEE in epilepsy centers are necessary to increase the diagnosis of genetic causes, adjust acute therapy, and minimize recurrence risk in future family planning.

## Conclusion

To sum up the analysis of neonatal seizures in a tertiary perinatal center in Germany just before the introduction of routine EEG monitoring shows that the consequent management of the highest clinical standard of care is crucial to lower the rate of acute provoked seizures further—in the preterm VLBW as well as in the term HIE patients. Neonatal EEG monitoring was already recommended as the standard for detecting neonatal seizures by the American Academy of Pediatrics, 2010 and has been implemented in most neonatal units since then but has become obligatory only after the time of our study. Moreover, the spectrum of seizures is broad, especially in sequential seizure types and the diagnostic yield of WES is crucial for recurrence risk prevention. Early detection of epileptic encephalopathies enables us to start targeted therapy at the earliest possible and improve developmental outcome of these patients as well as those with acute symptomatic lesions in the future.
